# Brain Mechanisms of Altered Consciousness in Focal Seizures

**DOI:** 10.3233/BEN-2011-0312

**Published:** 2011-03-29

**Authors:** Andrew P. Bagshaw, Andrea E. Cavanna

**Affiliations:** ^1^School of PsychologyUniversity of BirminghamBirminghamUK; ^2^Birmingham University Imaging CentreBirminghamUK; ^3^Department of NeuropsychiatryUniversity of Birmingham and BSMHFTBirminghamUK; ^4^Institute of NeurologyUCLLondonUK

**Keywords:** Epilepsy, consciousness, focal seizures, complex partial seizures, default mode network

## Abstract

Consciousness is a central concept in epileptology, relevant to the understanding of both focal and generalized seizures. Within focal seizures, impairment of consciousness has long been considered as the main criterion differentiating complex partial seizures (CPS) from simple partial seizures With the development of improved tools for investigating human brain function, new insights into the brain mechanisms of altered consciousness in CPS have become available. This paper reviews the existing literature on how the currently available methods can be used to address the fundamental issue of how CPS alter consciousness.

## 1. How is consciousness altered in complex partial seizures?

That consciousness is altered during complex partial seizures (CPS) can be clearly observed clinically and by patients and their carers, leading to impairment of consciousness being used as the major distinction between CPS and simple partial seizures (SPS) [11]. However, the answer to the question of exactly how consciousness is altered during CPS is complicated by the fundamental issue of defining what is meant by 'consciousness', a term that is more easily debated by philosophers than employed clinically by neurologists. What is often defined clinically as 'loss of consciousness' can be caused by much more specific disruptions of normal brain function. Standard clinical examination during and immediately after a CPS generally relies upon an assessment of the patient's responsiveness and ability to perform simple language, memory and motor tasks (e.g. response to verbal commands, object naming, reaching for an object etc.). Selective impairment of these systems as a result of primary or propagated seizure activity does not necessarily imply any alteration in the patient's level of consciousness, but the patient's unresponsiveness could easily be interpreted as such. Added to this definitional issue, the neuroscientific study of the brain mechanisms underlying consciousness is itself an active field, and one which is far from being resolved [8,12]. The available evidence suggests that consciousness is mediated by a distributed network of regions, collectively termed the 'neural correlates of consciousness' (NCC), which must be performing individually and collectively in order for consciousness to be preserved. Therefore, despite the intrinsic difficulty in defining what is precisely meant by consciousness, particularly in a neurological sense, the suggestion would be that alterations in consciousness during CPS occur when seizure activity develops from a focus and propagates to more extensive brain regions which are involved in the general maintenance of the conscious state.

However, while this provides a conceptual framework within which to examine the link between brain activity and behaviour, the exact mechanisms by which this disruption of the NCC occurs is not clear. With the

ISSN 0953-4180/11/$27.50 © 2011 – IOS Press and the authors. All rights reserved advent of improved tools for investigating human brain function, new insights into these issues are available, and the focus of this review will be on summarising how the available methods can be used to address the fundamental issue of how CPS alter consciousness. For example, recent studies have determined that the brain is intrinsically organised into a number of 'resting-state networks' (RSNs), often quite distributed sets of regions whose activity is correlated even in the absence of a task [14]. It seems that these networks, at least one of which has been linked with cognitive processes that are fundamental to consciousness, can be disrupted by epilepsy, even in the absence of a seizure [28,40]. With the application of these advanced neuroimaging techniques, allied with improvements in the way in which traditional tools such as intracranial EEG (iEEG) are analysed, a much more detailed understanding of the neurobiological mechanisms responsible for consciousness and its disruption by epilepsy is possible. However, to date, no single technique can provide the high spatiotemporal resolution coupled with access to activity from throughout the brain that would ideally be needed to study effects as widespread and complex as are likely to be responsible for alterations to consciousness. In addition, as mentioned above, standard clinical examination of a patient's state ictally and postictally does not provide detailed information about the nature of any alterations of consciousness. Tools such as the Ictal Consciousness Inventory, a twenty point questionnaire that can be used to examine the effect of CPS on the level and contents of consciousness, provide a much more specific way of identifying the precise behavioural effects of the seizure [9]. The level of consciousness depends on the integrity of the reticular activating system and thalamocortical pathways, while the contents of consciousness can be affected by disruption of more restricted systems, the precise details of which will depend on the focus of the patient's epilepsy. Detailed examination of the experiential effects of seizures, coupled with state-of-the-art neuroimaging techniques and analysis, not only has the potential to inform clinical management but also to serve as a tool for understanding the neural substrates of consciousness itself. Perhaps most importantly from a clinical viewpoint, if the underlying brain mechanisms responsible for these alterations in consciousness can be understood, the possibility of developing treatments to reduce the negative effects of seizures on patients' lives can be considered. Although clearly a complex problem, these goals provide the motivation to develop methods which can uncover the link between brain function and behaviour, and understand how a pathology as diverse as epilepsy can affect brain function.

## 2. How can the brain mechanisms of altered consciousness be investigated?

Like most other higher order brain functions, consciousness arises from the coordinated activity of a distributed network of cortical and subcortical regions. As such, the ideal method to identify the NCC (i.e. the minimum set of regions necessary for consciousness) would provide information from all regions of the brain with millimetre spatial and millisecond temporal precision [12]. Unfortunately, no such method currently exists, but the techniques that are available have been used to address some of the most important questions regarding the nature of the brain mechanisms that mediate altered consciousness in CPS. Although it is intuitively obvious that since the effects of interest occur during seizures, it is during seizures that patients should be studied, some very interesting observations have also been made interictally, both by observing the generators of interictal discharges and by examining the dynamics of the epileptic brain at rest.

The technique that has been most widely used in the investigation of CPS is single photon emission computed tomography (SPECT). SPECT involves the injection of a radionuclide (most commonly $^{99m}$Tc-HMPAO (hexamethylpropylene amine oxime), which gives images that are sensitive to cerebral blood flow) as soon as possible following the start of a seizure. The radionuclide very quickly becomes fixated in the brain tissues, with a regional distribution that is proportional to the blood flow, and hence the neuronal activity, at the time of injection. The patient is subsequently taken to the scanner following the end of the seizure, where the radioactive decay of the labelled compound is detected and reconstructed into an image which provides information about its distribution. The main advantages of SPECT are that it can be used ictally, and that it provides full brain sensitivity. The disadvantages are that it involves a considerable radiation dose, and so cannot be used repeatedly in individual subjects, and has a relatively low spatial and temporal resolution, meaning that the dynamic interactions between regions cannot be investigated.

At the other end of the temporal scale, EEG measures the coordinated post-synaptic activity of relatively widespread, synchronous neuronal populations with millisecond precision. When recorded non-invasively from the scalp the ability to identify the precise regions generating the signal is difficult, but during presurgical evaluation it is relatively common practice to record EEG data intracranially (iEEG) [8]. This provides a much more precise idea of the spatial location of the activity, as well as retaining the primary advantage of EEG, namely its high temporal resolution. There is increasing evidence that the coordination between regions is mediated by oscillatory electrical activity, meaning that examination of the EEG provides an excellent opportunity to determine the extent to which regions are functionally connected [35]. The disadvantage of iEEG is that the number of implanted electrodes is limited, and restricted to regions about which there is a clear clinical hypothesis, meaning that in practice there are limitations about the extent to which network activity can be assessed with iEEG. However, it can be an extremely powerful tool in selected patients.

Functional MRI (fMRI) has become one of the most widely used methods to study the human brain since its inception in the early 1990s. Its main advantage is that it has high spatial resolution and can provide whole brain coverage, meaning that it is an ideal tool to study the widespread, distributed networks that are often implicated particularly in higher order brain functions. However, it measures neuronal activity only indirectly via the haemodynamic response (HR), which also imposes a limit on the temporal resolution that can be achieved – the HR to even a very brief stimulus takes approximately five seconds to peak and does not return to its pre-stimulus level for another 10–15 seconds. Although fMRI has been used to examine the haemodynamic effects of seizures, the confined space available in the MRI scanner coupled with the relatively low likelihood of actually observing a seizure in the time available for scanning mean that interictal fMRI is more common [15,24,34]. Often this is combined with EEG to specifically examine interictal discharges [18], but another approach is to investigate spontaneous fluctuations of the fMRI signal across multiple brain regions, something for which fMRI is ideally suited. Of particular interest in this regard is the observation from the study of control subjects that the human brain is organised into a number of RSNs [14]. These RSNs can be identified by observing the correlations in the activity of the brain when it is not subjected to any task, and there is increasing evidence that these networks can be disrupted by epilepsy [28,40]. RSNs have been identified which link with intrinsic correlations of sensorimotor, visual, auditory, language and attention systems, and each seems to be related to a particular pattern of activity on the scalp EEG [29]. Perhaps most relevant to the current topic, a 'default mode network' (DMN) has been defined as consisting of regions in the frontal and parietal lobes, the cingulate gyrus and the thalamus [32]. This network of regions is generally deactivated whenever tasks requiring goal-directed attention are performed, and some of the cognitive processes that are fundamental to consciousness have been linked with activity in the DMN (e.g. conceptual processing, self-reflection) [10,20]. Interestingly, some of the regions that have been implicated in alterations of consciousness in patients with CPS overlap with those in the DMN [10,17].

Finally, although epilepsy and epileptic seizures are intrinsically features of abnormal brain function, information can also be extracted by investigating brain structure using MRI. Even when no obvious lesion can be detected by visual inspection as in the norm clinically, more sophisticated methods of quantitative analysis can sometimes identify subtle structural abnormalities, for example associated with abnormal signal intensity, cortical thickness etc [13]. In addition, new techniques such as diffusion tensor imaging (DTI) allow the underlying properties of the white matter connections between regions to be probed, which feasibly could be used to investigate the integrity of the links between the regions comprising the NCC [23].

With a topic as complex as consciousness, and the limitations of the available tools for investigating the human brain, it is perhaps not surprising that a full understanding of the mechanisms by which CPS can affect the level and contents of a patient's consciousness has yet to be formulated. However, considerable progress has been made, meaning that some firm conclusions can be drawn about how and why CPS lead to their observed behavioural effects.

## 3. What are the mechanisms of altered consciousness in CPS?

A seizure originating in and subsequently confined to the medial temporal lobe is likely to result in the disruption of processes subserved by those structures, in particular memory. This might lead to some ictal and postictal amnesia, but would be unlikely to result in other deficits. Thus, the observation that seizures originating from a focus can lead to alterations of consciousness (i.e. the observation that CPS exist) suggests that in certain circumstances focal seizures can affect more distant, and potentially more widespread, brain structures. The involvement of regions remote from the epileptogenic focus has been noted in a number of studies with various methodologies [5,26,31,36,38], but the precise relationship between this often widespread activity and alterations in consciousness remains unclear. What are the NCC, the regions necessary for the maintenance of the conscious state, and what are the neurobiological mechanisms by which they are disrupted during and after seizures?

The majority of the information about the regions that comprise the NCC, which are most straightforwardly identified during seizures, comes from SPECT studies. A number of these have suggested that CPS are associated with widespread, bilateral cortical and subcortical increases and decreases in blood flow [5, 36]. The most common pattern involves increases in the temporal lobe ipsilateral to the seizure focus, as well as bilateral subcortical midline structures, in particular the medial thalamus, basal ganglia and upper brainstem. In addition to these increases in blood flow (hyperperfusion), CPS demonstrate marked decreases in blood flow (hypoperfusion) in the bilateral orbitofrontal cortex, anterior and posterior cingulate regions and frontal and parietal association cortices. This pattern of increases and decreases in blood flow is specific to seizures which involve disruption of consciousness (i.e. CPS), with SPS showing much more limited changes primarily involving the temporal lobe itself [5]. These regions, then, could be characterised as the NCC. By analysing signal correlations across the different activated and deactivated regions, it is possible to get an idea of the how they are connected, leading to the observation that fronto-parietal hypoperfusion is correlated with midline subcortical structures, including the thalamus [5]. This has lead to the 'network inhibition hypothesis' [4, 16], which suggests that propagation of seizure activity to subcortical structures that are needed for cortical activation disrupts their normal function and consequently the cortex is deactivated, entering a state that may have similarities with sleep. Support for such a hypothesis comes from the observation of slow wave activity, which is also observed in sleep, in the thalamus and fronto-parietal regions during CPS [6,22].

While SPECT is able to localise the regions affected during CPS, and hence to identify the network that forms the NCC, its ability to investigate the functional connections between these regions is limited by its low temporal resolution. Given that the activity within a network must be temporally coordinated if the network is to function correctly, a direct assessment of how regions are coordinated is likely to shed light on how they might become disrupted as a result of seizures. Two main approaches have been taken to address this issue, namely iEEG and fMRI. As discussed above, iEEG has ideal temporal resolution and is also a relatively unambiguous measure of brain activity, compared to the haemodynamic changes that form the basis of the fMRI signal. However, the electrodes are placed purely on clinical grounds, and there are relatively few of them, meaning that the activity of regions involved in the NCC cannot always be measured. Despite this, in selected cases there may be clinical reasons for implanting a relatively large number of electrodes, and if the trajectory of an electrode is opportune it may still be possible to record from some structures of interest, even if they are not the target. In one example of this, Guye et al. [22] were able to select 13 TLE patients with electrode contacts in the cortex and the thalamus from a total of 82 who had undergone iEEG. This allowed them to study thalamocortical coupling during CPS and to observe that patients with mesial TLE (mTLE) who experienced early loss of consciousness during CPS also had a higher degree of thalamocortical synchrony. The idea that abnormal thalamocortical synchrony might have a role in alterations of consciousness during CPS was supported by more recent work from the same group, which found that increased synchrony in structures outside of the temporal lobe was strongly correlated with the degree to which consciousness was altered [2].

MRI techniques can also be used to shed light on these issues. In particular, as discussed above, fMRI is ideally suited to the study of widespread networks and the connectivity between the individual regions of the network. Of particular relevance, it is becoming increasingly widely accepted that the DMN is disrupted in both generalised and partial epilepsy. The DMN, originally identified from the observation of common regions of deactivation across multiple tasks, is posited to represent baseline brain activity related to self-oriented mental activity [20,32]. Regions associated with the DMN have been observed to be deactivated in response to interictal epileptic discharges (IEDs) in generalised [1,19] and partial [25,27,33,39], suggesting a mechanism whereby interictal discharges may disrupt normal brain function. In addition, even in the absence of IEDs, the spontaneous activity of the DMN has been observed to be disrupted in TLE [40]. With its relatively good temporal resolution, fMRI can be used to explicitly probe the relationship between the signal fluctuations in different regions (i.e. functional connectivity). This has indicated that patients with mesial TLE have increased connectivity within the temporal lobes compared to control subjects, but decreased connectivity in fronto-parietal networks [28]. A similar conclusion regarding the functional connectivity of the temporal lobes has also been reached via iEEG [3], and analysis of structural MRI data also points to abnormal structural connectivity in patients with partial epilepsy [13]. Advanced mathematical tools such as graph theory allow the degree of connectivity between the nodes of a network to be explicitly quantified [38], and may in the future provide a framework to characterise and summarise the alterations to connectivity caused by epilepsy [21,28]. One of the advantages of graph theoretical analysis is that it is able to identify particularly well-connected regions of a network, so called 'hubs'. Interestingly, several studies have identified the precuneus/posterior cingulate region as a hub region, consistent with its high baseline metabolic rate and seemingly important position within the DMN [32]. Such observations, linked with the previous discussion about the regions involved in the NCC, may suggest that the involvement of the posterior cingulate region is particularly important in determining whether a seizure will result in alterations of consciousness [10]. A study into the functional connectivity of generalised spike-wave discharges, which can also lead to loss of consciousness, similarly suggested that the precuneus is fundamentally important [37], although similar analyses have yet to be performed in partial epilepsy. The development of these analysis tools, which can be explicitly used to probe how spatially distributed regions coordinate their activity, has considerable potential to enable non-invasive neuroimaging techniques to be used to understand distributed network processes.

## 4. Conclusions

The available evidence from multiple methods of investigation provides a coherent picture as to how activity from focal seizures is able to lead to global disruptions in consciousness. Within this framework, seizure activity spreads from its site of origin into wider brain regions, causing abnormally enhanced synchrony in regions responsible for the maintenance of the conscious state, which may serve to mimic the pattern more normally seen during sleep. A widespread network of regions has been identified which are necessary for consciousness, involving bilateral subcortical midline structures, in particular the medial thalamus, basal ganglia and upper brainstem, as well as bilateral orbitofrontal cortex, anterior and posterior cingulate regions and frontal and parietal association cortices. Several of these regions overlap with the DMN, and the observation that patients with partial epilepsy have modified resting state brain activity, particularly in the DMN, suggests that there is an inherent modification of normal thalamocortical connections, potentially because of the cumulative effect of seizures. The seizure activity may then be able to take advantage of these connectional anomalies to interfere with DMN activity, leading to the clinical observation of a CPS. However, the precise nature of the relationship between the regions of the NCC and the DMN remains unclear, making this a plausible though unproven hypothesis. A further complication is that almost all of the studies in partial epilepsy have been conducted in patients with mTLE, and although similar mechanisms would be expected to occur in other CPS, the data to support this is sparse.

Within this general framework, several lines of investigation remain for future studies. Although resting fMRI studies are relatively straightforward to perform, and connect with a considerable literature from control subjects, their link with ictal SPECT and iEEG results in the same patients remains to be clarified. Similarly, although there are several tools to characterise the nature of ictal alterations in consciousness, they are often not used in neuroimaging studies, meaning that a precise and detailed understanding of the behavioural effects of seizures in specific patients is not available. New methods of connectivity analysis such as graph theory may help to quantify the nature of the disruptions caused by epilepsy, and can be used to identify particularly important regions within a network. With these developments leading to more precise understanding of the neural mechanisms underlying alterations to consciousness in CPS, the next step will be to identify ways in which this spread of activity can be disrupted and consciousness retained.
